# A comprehensive classification and nomenclature of carboxyl–carboxyl(ate) supramolecular motifs and related catemers: implications for biomolecular systems

**DOI:** 10.1107/S205252061500270X

**Published:** 2015-03-24

**Authors:** Luigi D’Ascenzo, Pascal Auffinger

**Affiliations:** a Architecture et Réactivité de l’ARN, Université de Strasbourg, Institut de Biologie Moléculaire et Cellulaire du CNRS, 67084 Strasbourg, France

**Keywords:** supramolecular motifs, biomolecular systems, crystal engineering, pharmaceuticals

## Abstract

Carboxyl and carboxylate groups form important supramolecular motifs (synthons). Besides carboxyl cyclic dimers, carboxyl and carboxylate groups can associate through a single hydrogen bond. Carboxylic groups can further form polymeric-like catemer chains within crystals. To date, no exhaustive classification of these motifs has been established. In this work, 17 association types were identified (13 carboxyl–carboxyl and 4 carboxyl–carboxylate motifs) by taking into account the *syn* and *anti* carboxyl conformers, as well as the *syn* and *anti* lone pairs of the O atoms. From these data, a simple rule was derived stating that only eight distinct catemer motifs involving repetitive combinations of *syn* and *anti* carboxyl groups can be formed. Examples extracted from the Cambridge Structural Database (CSD) for all identified dimers and catemers are presented, as well as statistical data related to their occurrence and conformational preferences. The inter-carboxyl(ate) and carboxyl(ate)–water hydrogen-bond properties are described, stressing the occurrence of very short (strong) hydrogen bonds. The precise characterization and classification of these supramolecular motifs should be of interest in crystal engineering, pharmaceutical and also biomolecular sciences, where similar motifs occur in the form of pairs of Asp/Glu amino acids or motifs involving ligands bearing carboxyl(ate) groups. Hence, we present data emphasizing how the analysis of hydrogen-containing small molecules of high resolution can help understand structural aspects of larger and more complex biomolecular systems of lower resolution.

## Abstract 2

The vast diversity of carboxyl–carboxyl(ate) arrangements is reduced to 17 supramolecular motifs and eight catemers. Examples of each, extracted from the CSD, are presented.

## 1. Introduction   

Carboxyl and carboxylate [written collectively as carboxyl(ate)] groups are found in a large variety of biomolecular compounds and also in drugs and synthetic molecular systems. For the former, the two Asp and Glu amino acids represent ∼ 2% of the ∼ 2 million amino acids found in the Protein Data Bank (PDB, November 2014 release; Berman *et al.*, 2000 ▶). For the latter, they assemble to form essential supramolecular synthons recurrently used in crystal engineering (Desiraju, 2007 ▶, 2013 ▶; Merz & Vasylyeva, 2010 ▶) and are present in ∼ 37 000 (∼ 5–6%) of the ∼ 675 000 crystal structures in the Cambridge Structural Database (CSD Version 5.35, November 2013; see Table 1 ▶; Allen, 2002 ▶; Chisholm *et al.*, 2006 ▶; Groom & Allen, 2014 ▶).

Despite the fact that carboxyl groups figure among the best investigated hydrogen-bond functionalities (Huggins, 1936 ▶; Leiserowitz, 1976 ▶; Berkovitch-Yellin & Leiserowitz, 1982 ▶; Steiner, 2001 ▶, 2002 ▶; Das & Desiraju, 2006 ▶; Rodríguez-Cuamatzi *et al.*, 2007 ▶), no systematic classification of carboxyl–carboxyl motifs is currently available. This is also true, but to a lesser extent, for carboxyl–carboxylate interaction modes (Wohlfahrt, 2005 ▶; Rodríguez-Cuamatzi *et al.*, 2007 ▶; Langkilde *et al.*, 2008 ▶). Indeed, the latter interaction types are essential in biology where numerous close contacts between Asp/Glu side chains have been reported (Gandour, 1981 ▶; Sawyer & James, 1982 ▶; Ramanadham *et al.*, 1993 ▶; Flocco & Mowbray, 1995 ▶; Torshin *et al.*, 2003 ▶; Wohlfahrt, 2005 ▶; Langkilde *et al.*, 2008 ▶).

**Figure 1:**
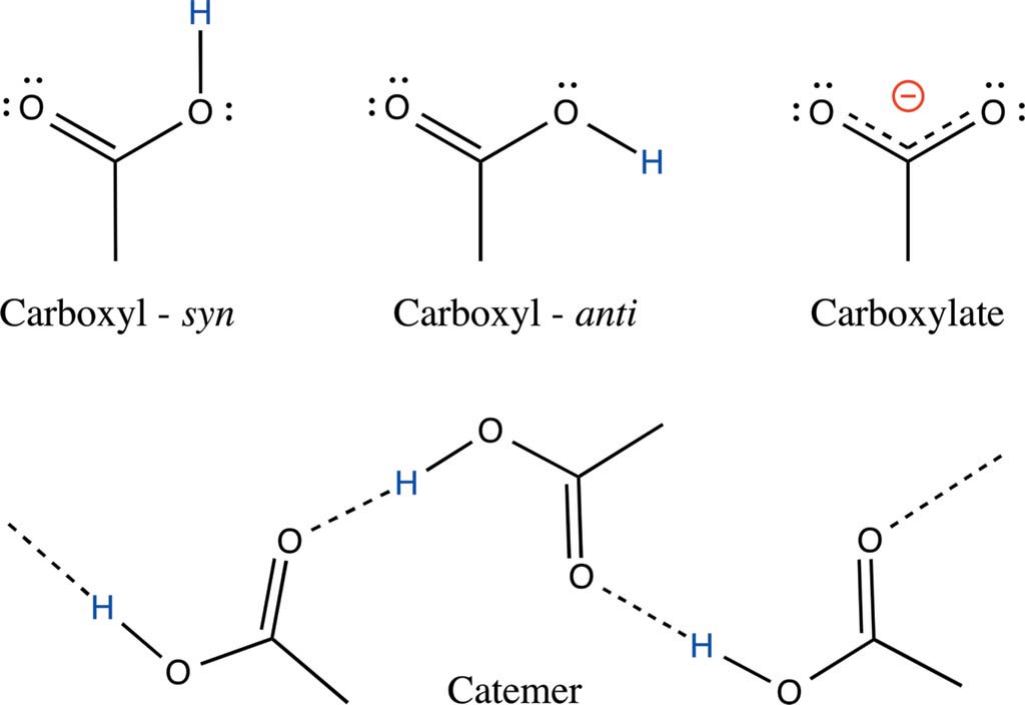
Carboxyl(ate) groups (*syn* and *anti* conformers) and schematic structure of a catemeric chain. The *syn* and *anti* lone pairs of the three carboxyl(ate) O atoms are marked by double dots.

For synthetic carboxyl dimers, the most common interaction mode is the centrosymmetric cyclic dimer, but numerous other dimers involving a single interlinking hydrogen bond have been characterized. Interestingly, some of these dimers can form catemers (Fig. 1 ▶), defined as infinite one-dimensional patterns involving their carboxyl groups (Leiserowitz, 1976 ▶; Berkovitch-Yellin & Leiserowitz, 1982 ▶; Kuduva *et al.*, 1999 ▶; Beyer & Price, 2000 ▶; Das *et al.*, 2005 ▶; Das & Desiraju, 2006 ▶; DeVita Dufort *et al.*, 2007 ▶; Rodríguez-Cuamatzi *et al.*, 2007 ▶; Saravanakumar *et al.*, 2009 ▶; Sanphui *et al.*, 2013 ▶). A complete classification of catemer motifs is also currently missing.

The formation of carboxyl(ate) dimers and further of carboxyl catemer motifs implies the involvement of common *syn* but also less common *anti* conformers, as well as the *syn* and/or *anti* lone pairs of the O atoms (Görbitz & Etter, 1992*a* ▶; Das *et al.*, 2005 ▶; Das & Desiraju, 2006 ▶; Sanphui *et al.*, 2013 ▶; Fig. 1 ▶). Theoretical studies have investigated the relative stability of the *syn* and *anti* conformers. It is generally accepted that in the gas phase, the *syn* conformer is favoured over the *anti* conformer by 21.4–28.9 kJ mol^−1^ depending on the theoretical level and basis set used in quantum chemical calculations (Kamitakahara & Pranata, 1995 ▶; Sato & Hirata, 1999 ▶; Nagy, 2013 ▶). In aqueous solution, the estimated relative energy difference between the two conformers is reduced to 7.12 kJ mol^−1^ (Nagy, 2013 ▶). A further point of interest involves the relative basicity of the *syn* and *anti* lone pairs of carboxylate groups. Theoretical studies have reported that although the *syn* lone pairs are intrinsically more basic, the basicity difference decreases and even cancels out when environmental effects are taken into consideration (Li & Houk, 1989 ▶; Allen & Kirby, 1991 ▶; Gao & Pavelites, 1992 ▶). In line with these data, a significant number of catemer motifs involving *anti* conformers have been observed in various crystallographic surroundings, supporting the fact that environmental effects are able to reverse anticipated conformational equilibria (Das & Desiraju, 2006 ▶). *Anti* conformers have also been considered in drug discovery strategies involving bioisosterism (McKie *et al.*, 2008 ▶; Meanwell, 2011 ▶; Allen *et al.*, 2012 ▶).

Given the importance of these carboxyl–carboxyl(ate) dimers in both the chemical and biochemical realms, the present study aims at:

(i) providing an exhaustive classification of all possible dimers and catemers involving these groups;
(ii) proposing a systematic nomenclature for them;
(iii) defining recurrent hydrogen-bond properties.

This study should contribute to an improved understanding of the structural diversity observed in small-molecule crystal structures, and provide insights into crystal engineering of new materials (Desiraju, 2007 ▶, 2013 ▶), including pharmaceutical co-crystals (Blagden *et al.*, 2007 ▶). However, the main incentive of the study resides in acquiring reliable statistical data that will help to understand carboxyl(ate) interactions in biomolecular systems. In this respect, analysing small-molecule crystal structures, where H atoms are systematically observed, has a clear edge over exploring biomolecular systems where H atom positions are rarely reported (Ahmed *et al.*, 2007 ▶; Fisher *et al.*, 2012 ▶).

## 2. Methods   

The Cambridge Structural Database (CSD Version 5.35, November 2013) was searched for structures containing carboxyl–carboxyl(ate) motifs by using explicit H-atom positions. All searches were performed with the *ConQuest* software (Bruno *et al.*, 2002 ▶) using filters so that error-containing, polymeric and powder structures were excluded, as well as structures marked as disordered. Although H-atom disorder is common in carboxylic systems, structures where the H atom could not be unambiguously assigned to a single O atom were not considered (Leiserowitz, 1976 ▶; Berkovitch-Yellin & Leiserowitz, 1982 ▶; Wilson *et al.*, 1996 ▶; Das *et al.*, 2005 ▶; Thomas *et al.*, 2010 ▶; Hursthouse *et al.*, 2011 ▶). This criterion leads to exclusion of 12 out of the 23 catemers listed by Das & Desiraju (2006 ▶). However, Steiner (2001 ▶) reported that statistics were not affected by excluding disordered structures. The searches were also restricted to structures with low *R*-factor values (*R* ≤ 0.05) unless otherwise specified. Metal-bound carboxyl(ate) groups were excluded given their specific structural features (Hocking & Hambley, 2005 ▶). Note that the November 2006 CSD release contained less than 2/3 of the structures found in the November 2013 release. Thus, the present searches significantly extend those presented in earlier publications on smaller samples of structures (Kuduva *et al.*, 1999 ▶; DeVita Dufort *et al.*, 2007 ▶; Langkilde *et al.*, 2008 ▶).

Since carboxyl(ate) groups are involved in strong hydrogen bonds (Jeffrey, 1997 ▶; Steiner, 2001 ▶; Langkilde *et al.*, 2008 ▶), a stringent hydrogen-bond cut-off criterion could be used (O⋯O ≤ 2.8 Å). The H-atom positions were not considered for analysing hydrogen-bond lengths since their position is systematically unreliable when not derived from neutron diffraction experiments (Vishweshwar *et al.*, 2004 ▶; Allen & Bruno, 2010 ▶). Neutron diffraction surveys provide an average 1.018 Å (Allen & Bruno, 2010 ▶) or even a 1.070 Å value (Vishweshwar *et al.*, 2004 ▶) for the carboxyl O—H bond length, compared with an average of 0.87 Å derived from our survey. Hence, we have not used H atoms in the subsequent analysis, except for obviously differentiating carboxyl from carboxylate groups and for defining the *syn*/*anti* character of the former. An incidental advantage of not using H atoms is that our defined criteria can be used in biomolecular systems where H atoms are rarely characterized.

The geometric parameters used to distinguish the *syn* and *anti* conformers of the carboxyl groups and the spatial *syn* and *anti* arrangement of carboxyl–carboxyl(ate) dimers are detailed in §3.1. Specific criteria were used to exclude a few borderline and possibly error-containing structures. For instance, the WEGBUH structure (Ying, 2012 ▶) contains a short (2.58 Å) interaction between two O atoms of the carboxylic hydroxyl groups that corresponds rather to a carboxyl–carboxylate than to a carboxyl–carboxyl motif. Similarly, a significant number of structures are excluded where the H atoms are located out of the O=C—O plane by more than 0.4 Å.

The results of the searches were analysed using *Vista* (CCDC, 1994 ▶), and all structures were visualized using *Mercury* CSD Version 3.3 (Macrae *et al.*, 2008 ▶). Catemer structures were individually examined and classified. The possibility that some of the presented catemer motifs could belong to large rings rather than infinite chains was considered and excluded.

## 3. Results   

### 3.1. Carboxyl and carboxylate groups   

**Figure 2:**
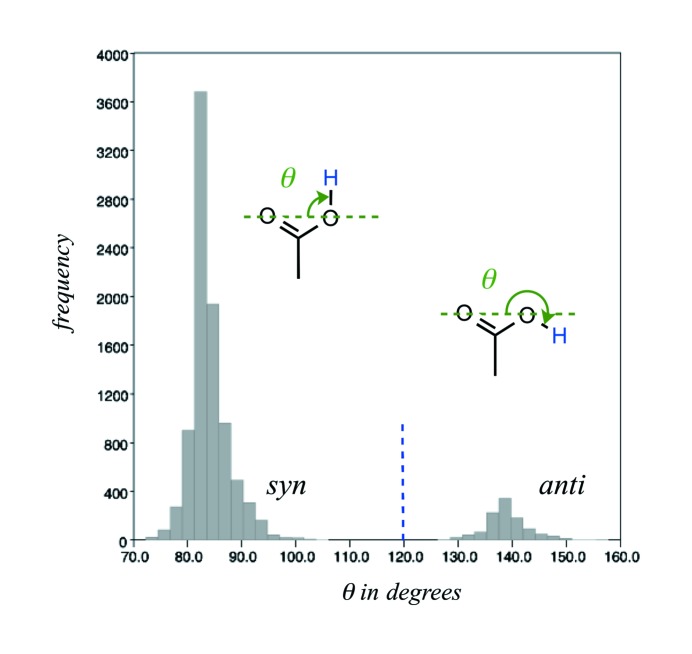
Geometric parameters used for separating the carboxyl *syn* and *anti* conformers. The *syn* conformers are defined by a θ value below 120° (marked by a blue dashed line on the histogram; θ corresponds to the O⋯O—H angle). The *anti* conformers are defined by a θ value greater than 120°. The histogram has been derived from an ensemble of low *R*-factor (*R* ≤ 0.05) carboxylic acid containing structures.

Carboxylic acids bear a proton that is commonly found in the *syn* and more rarely in the *anti* conformation. In order to distinguish between the *syn* and *anti* conformers, we imposed the following criterion on the O⋯O—H angle (θ) (Fig. 2 ▶). The *syn* conformer corresponds to θ angle values between 0 and 120°; the *anti* confirmer to θ angle values between 120 and 180°. The relative proportion of these conformers is roughly 9/1 in favour of *syn*, while negatively charged carboxylate groups represent about 2/3 of the total carboxyl groups (Table 2 ▶). The main geometric features of carboxyl(ate) groups are similar to those reported in an early study (Leiserowitz, 1976 ▶). Our updated values are reported in Table 2 ▶. Note that, due to its partial double-bond character, the C=O bond of carboxyl groups is shorter by ∼ 0.11 Å than the adjacent C—O(H) hydroxyl bond.

**Figure 3:**
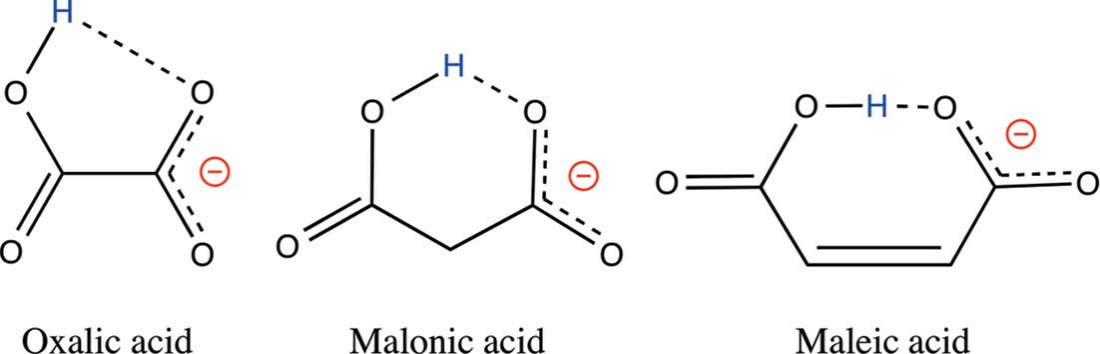
Three dicarboxylic acids with an *anti* carboxyl group involved in an intramolecular hydrogen bond, schematically displayed under the CSD most represented mono-anion dicarboxylic acid form.

The *anti* conformer population is more heterogeneous than the *syn* population since they are involved in a large diversity of intermolecular but also intramolecular bonds such as those observed in oxalic, malonic, maleic (Fig. 3 ▶) as well as phthalic acids. For the three former acids in their most represented mono-anion dicarboxylic acid form, the average *d*(O⋯O) hydrogen-bond distances are 2.67 ± 0.03 (10 structures), 2.46 ± 0.03 (20 structures) and 2.44 ± 0.03 Å (107 structures), stressing the formation of very short hydrogen bonds. Since the scope of this study is to examine supramolecular motifs, we eliminated from our searches all ‘intramolecular’ contacts involving an *anti* carboxyl conformer unless otherwise specified. When structures containing intramolecular hydrogen bonds were excluded, the number of fragments containing an *anti* carboxyl conformer decreased from 1168 to 223.

### 3.2. Carboxyl–carboxyl(ate) interactions   

#### 3.2.1. Nomenclature   

**Figure 4:**
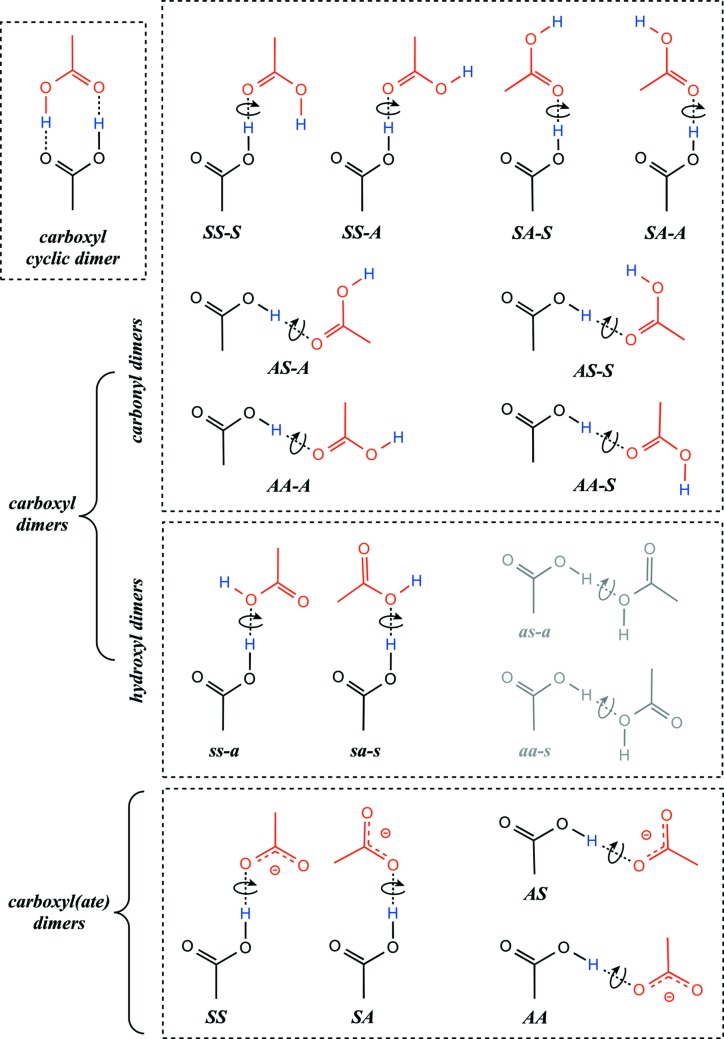
All 17 possible carboxyl–carboxyl(ate) dimers with accompanying nomenclature. The cyclic dimer is represented in the top left box; the eight ‘*carbonyl dimers*’ involving a hydroxyl donor and a carbonyl acceptor group are represented in the top right box; the four ‘*hydroxyl dimers*’ involving a donor and acceptor hydroxyl group are represented in the central box (the two *as-a* and *aa-s* dimers not identified in the CSD are shaded); the four carboxyl–carboxylate dimers are represented in the bottom box.

An evaluation of carboxyl(ate) interaction modes based on the *syn*/*anti* carboxylic conformers and the *syn*/*anti* carboxyl(ate) lone pairs led to a total of 17 carboxyl–carboxyl(ate) dimers comprising: (i) one cyclic dimer; (ii) 12 carboxyl–carboxyl dimers involving a single hydrogen bond; (iii) 4 carboxyl(ate) dimers. Free rotation around the interlinking hydrogen bond is considered for all except the cyclic dimer (Fig. 4 ▶). The formation of three-centred or bifurcated hydrogen bonds was not considered since they do not appear in previous (Görbitz & Etter, 1992*b* ▶) and current CSD surveys as well as in molecular dynamics simulations of formate and acetate ions in water (Payaka *et al.*, 2009 ▶, 2010 ▶). This simplifies considerably the presented nomenclature.

Sixteen interaction modes involve a single hydrogen bond linking the two units. We propose a three letter nomenclature for carboxyl–carboxyl dimers based on:

(i) the *syn* or *anti* conformer of the first carboxyl group that is by convention always the hydrogen-bond donor group of the dimer;
(ii) the *syn* or *anti* lone pair of the carbonyl hydrogen-bond acceptor group of the dimer;
(iii) the *syn* or *anti* conformer of the dimer hydrogen-bond acceptor group.

The first letter (*S* or *A*) corresponds to the *syn* or *anti* conformer; the second letter (*S* or *A*) to the lone pair involved in the hydrogen bond; the third letter (*S* or *A* separated by a dash from the two others) to the position of the H atom not involved in the hydrogen bond. For the eight dimers involving the participation of a carbonyl lone pair in the hydrogen bond (‘*carbonyl dimers*’), capital letters are used. Lowercase letters are used for the four dimers involving the hydroxyl lone pair (‘*hydroxyl dimers*’). A two capital-letter code suffices for the four carboxyl–carboxylate dimers.

#### 3.2.2. Geometric classification criteria   

**Figure 5:**
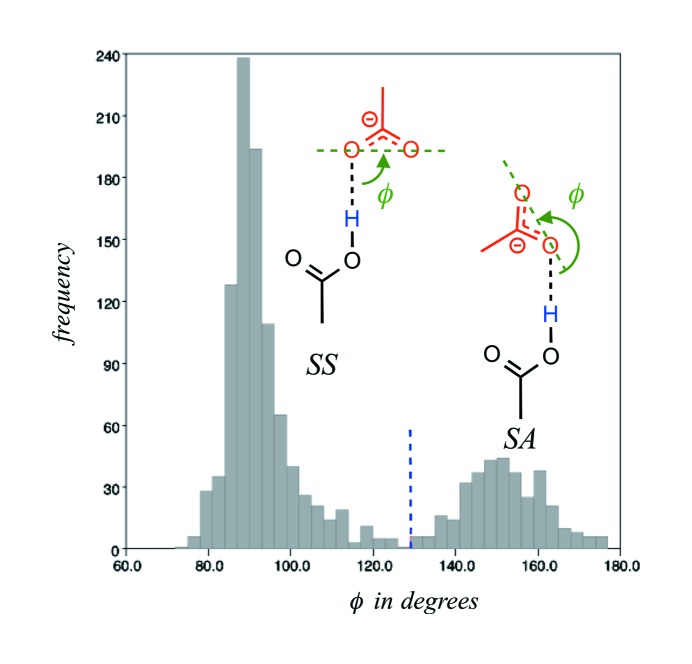
Geometric parameters used for separating carboxyl–carboxylate dimers involving *syn* or *anti* lone pairs. The histogram has been drawn for a sub-ensemble of *SS* and *SA* dimers. The *syn* conformers are defined by a φ value below 130° marked by a blue dashed line on the histogram; φ corresponds to the O(H)⋯O⋯O angle. The *anti* conformers are defined by a φ value greater than 130°.

As noted above (Fig. 2 ▶), simple geometric criteria can be used to filter the carboxyl *syn* and *anti* conformers. It was less obvious how to discriminate dimers based on their *syn* or *anti* lone pair bonding types. After having tried several options, we found that the histograms showing the φ angle that corresponds to the O(H)⋯O⋯O angle involving the hydrogen-bond donor O atom and the two carboxylate O atoms are the most helpful to achieve such a goal. The histogram drawn for the carboxyl–carboxylate dimers is unambiguous and prompted us to use a 130° cut-off for isolating the *SS* and *AA* from the *SA* and *AS* carboxyl–carboxylate dimers, respectively (Fig. 5 ▶). Although a clear partition is difficult to identify on the *SS-S* dimer histogram (data not shown), a visualization of these dimers confirmed the soundness of the defined criteria. As is often the case, borderline conformations are observed and are difficult to eliminate but do not alter the inferred landscape.

#### 3.2.3. Carboxyl–carboxyl interaction modes   

**Figure 6:**
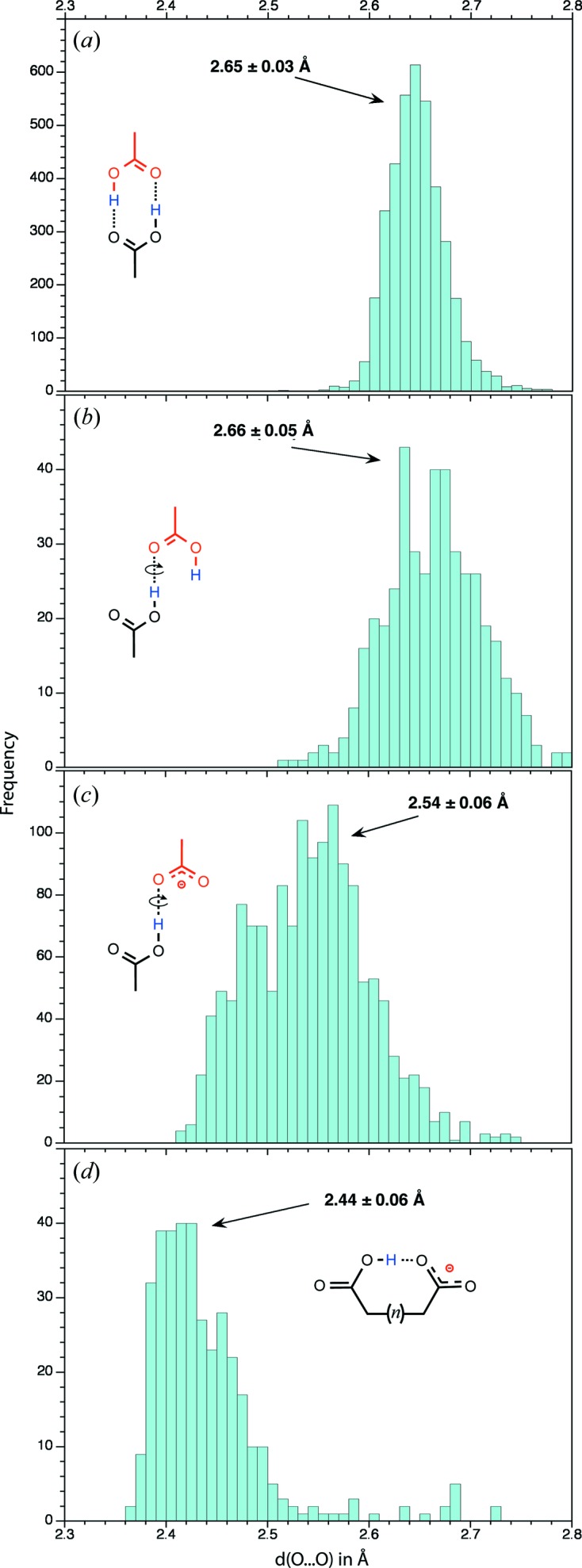
Histograms showing the distance distribution between the two O atoms involved in the interlinking hydrogen bond(s) for carboxyl–carboxyl(ate) dimer structures with low *R*-factors (*R* ≤ 0.05). The arrows mark the average values. (*a*) *d*(O⋯O) histogram for the two carboxyl⋯carboxyl hydrogen bonds of the cyclic dimers. (*b*) *d*(O⋯O) histogram for the non-cyclic carboxyl⋯carboxyl hydrogen bonds. All *syn* and *anti* conformers are taken into account. (*c*) *d*(O⋯O) histogram for the carboxyl⋯carboxylate hydrogen bonds (intramolecular hydrogen bonds are not considered). All *syn* and *anti* conformers are taken into account. (*d*) *d*(O⋯O) histogram for the carboxyl⋯carboxylate intramolecular hydrogen bond found in mono-anion dicarboxylic acids (see for instance Fig. 3 ▶).

*Cyclic dimer*: This dimer is undoubtedly the best represented in the CSD (Table 3 ▶). The distance between the O atoms involved in the hydrogen bond is on average close to 2.65 ± 0.03 Å (Fig. 6 ▶) and consequently shorter by 0.17 Å than the accepted H_2_O⋯OH_2_ hydrogen-bond length (2.82 Å). Cyclic dimers are almost perfectly planar.

**Figure 7:**
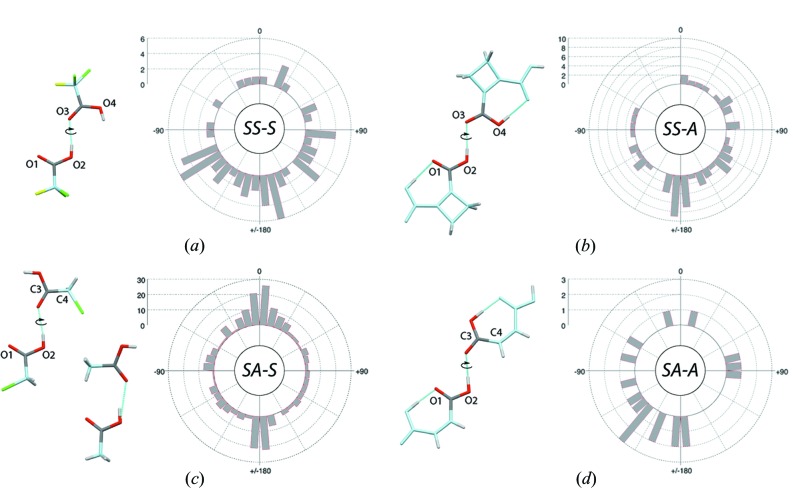
Carboxyl–carboxyl dimers involving a *syn* conformer and the lone pair of a carbonyl group (‘*carbonyl dimer*’) along with their rotamer distribution around the interlinking hydrogen bond for structures with *R* ≤ 0.05. The C and O atoms not belonging to the interacting carboxyl groups are shown in light blue, F and Cl atoms are shown in yellow and green, respectively. (*a*) *Antiplanar SS-S* dimer (NAGVUM) and O1—O2—O3—O4 dihedral angle rotamer histogram. (*b*) *Antiplanar SS-A* dimer (CBUCDX01) and O1—O2—O3—O4 dihedral angle rotamer histogram. (*c*) *Antiplanar* and *synplanar SA-S* dimers (CLACET01 and ACETAC09) and O1—O2—C3—C4 dihedral angle rotamer histogram. (*d*) *Antiplanar SA-A* dimer (MALIAC12) and O1—O2—C3—C4 dihedral angle rotamer histogram.

‘*Carbonyl dimers*’: Eight ‘*carbonyl dimer*’ types were identified (Table 3 ▶). The four types involving the *syn* conformer of the donor carboxyl group and among them, the *SA-S* dimers, are well represented. The *synplanar* rotamers are generally not observed except for the *SA-S* dimers where they are as prominent as *antiplanar* rotamers (Fig. 7 ▶). Note that *syn*- and *antiplanar* rotamers are defined by inter-dimer dihedral angles with values close to 0 and 180°, respectively (see, for example, Fig. 7 ▶ *c*). The ACETAC09 acetic acid structure seems to be stabilized by a C—H⋯O interaction involving the methyl group, an orientation that is not found for chloroacetic acid in the CLACET01 structure and illustrates how weak interactions participate in structural networks.

**Figure 8:**
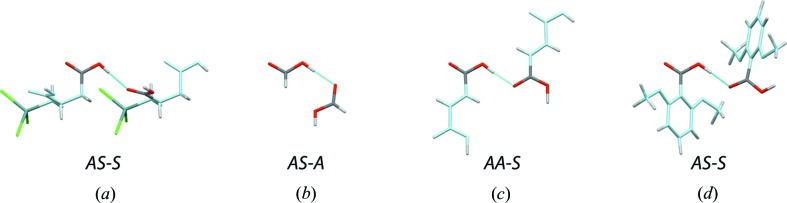
Carboxyl–carboxyl dimers involving an *anti* conformer and the lone pair of a carbonyl group (‘*carbonyl dimer*’). The C and O atoms not belonging to the interacting carboxyl groups are shown in light blue, Cl and Ge atoms are shown in green and dark green, respectively. (*a*) *AS-S* dimer (WOKPOC). (*b*) *AS-A* dimer (NEWXAO). (*c*) *AA-S* dimer involving two fumaric acid molecules (KACNAD). (*d*) *AA-A* dimer (DMOXEA01).

Not surprisingly, the four dimer types involving the *anti* conformer of the donor carboxyl are rare. Among them, the *AA-S* dimer that involves the *anti* lone pair of a carbonyl group is best represented. However, convincing structures are found for each dimer type (Fig. 8 ▶). The hydrogen-bond length distribution is broader than the one given for the cyclic dimers, while the average hydrogen-bond length is roughly the same (2.66 ± 0.05 Å; Fig. 6 ▶).

**Figure 9:**
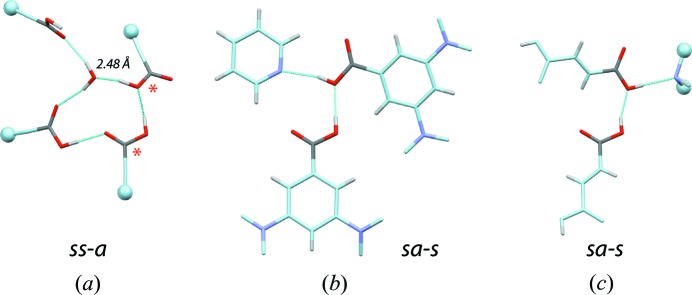
Rare carboxyl–carboxyl dimers involving the lone pair of the hydroxyl group (‘*hydroxyl dimers*’). The C and O atoms not belonging to the interacting carboxyl groups are shown in light blue, N atoms are shown in magenta. The light blue spheres indicate that the molecule has been truncated for visualization purposes. (*a*) *Antiplanar SS-A* dimer (CACTUW; *R* = 0.04). Due to the size of the system, only the interacting fragments are shown. The unusually short carboxyl–O*w* distance is given. The red asterisks mark the carboxyl groups involved in the *ss-a* dimer. (*b*) *Antiplanar sa-s* dimer (CAYJAO; *R* = 0.06). (*c*) *Synplanar sa-s* dimer involving two fumaric acid molecules (EMONAW; *R* = 0.11). The N-containing interacting molecule has been truncated due to its size.

‘*Hydroxyl dimers*’: Although the two carboxyl hydroxyl groups could form hydrogen bonds, this interaction occurs rarely. Only two *ss-a* and six *sa-s* conformers were characterized (Table 3 ▶; Fig. 9 ▶). None of the two other possible *as-a* and *aa-s* conformers were observed. This points to the fact that the lone pairs of carboxyl —OH groups seem to be much less basic and/or accessible to other carboxyl groups than the lone pairs of more common hydroxyl groups.

#### 3.2.4. Carboxyl–carboxylate interaction modes   

**Figure 10:**
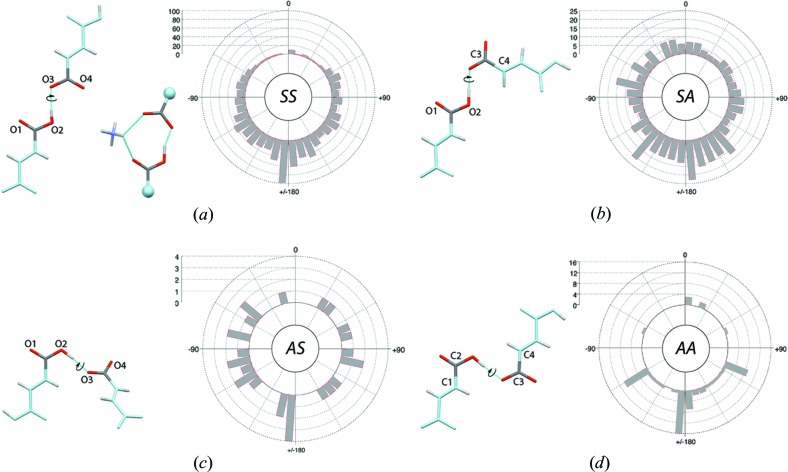
The four carboxyl–carboxylate dimer types and their rotamer distribution around the interlinking hydrogen bond for structures with *R* ≤ 0.05. The C and O atoms not belonging to the interacting carboxyl or carboxylate groups are shown in light blue, N atoms are shown in magenta. (*a*) (Left) *Antiplanar SS* dimer involving two fumaric acid molecules (HUSSUJ). (Middle) *Synplanar SS* dimer (JEDPUE). An NH_4_^+^ molecule links the carboxyl(ate) groups. The light blue spheres indicate that the molecule has been truncated for visualization purposes. (Right) O1—O2—O3—O4 dihedral angle rotamer histogram. (*b*) *Antiplanar SA* dimer involving two fumaric acid molecules (CLEMAS) and O1—O2—C3—C4 dihedral angle rotamer histogram. (*c*) *Antiplanar AS* dimer involving two fumaric acid molecules (SEGSAZ) and O1—O2—O3—O4 dihedral angle rotamer histogram. (*d*) *Antiplanar AA* dimer involving two fumaric acid molecules (BAHLEC) and C1—C2—C3—C4 dihedral angle rotamer histogram.

The *SS* dimer, involving a hydrogen bond between a *syn* hydroxyl group and a *syn* carboxylate lone pair, is the most prevalent carboxyl–carboxylate dimer in the CSD (Table 3 ▶). The *antiplanar SS* dimer is frequently observed while dimers close to the *synplanar* orientation are much less represented (Fig. 10 ▶). Some rare occurrences of the *synplanar* orientation stabilized by intervening groups (such as NH_4_^+^ in JEDPUE; see Fig. 10 ▶) are reported. In those instances, the distances between the O atoms not involved in the hydrogen bond exceed 3.0 Å.

All *SA* rotamers, involving a hydrogen bond between a *syn* hydroxyl group and an *anti* carboxylate lone pair, are nicely represented with some preference for the *antiplanar* orientations. The *AS* and *AA* dimers are less abundant but are still observed in a significant number of structures.

The most distinctive feature of these carboxyl–carboxylate dimers is related to the very short average hydrogen-bond distance between the two O atoms (2.54 ± 0.06 Å), which does not seem to be dependent on the dimer type (Fig. 6 ▶). The shortest observed hydrogen bonds (2.43 ± 0.04 Å) belong to intramolecular mono-anion dicarboxylic acids (Figs. 6 ▶ and 10 ▶).

#### 3.2.5. Carboxyl(ate)–water hydrogen-bond length   

**Figure 11:**
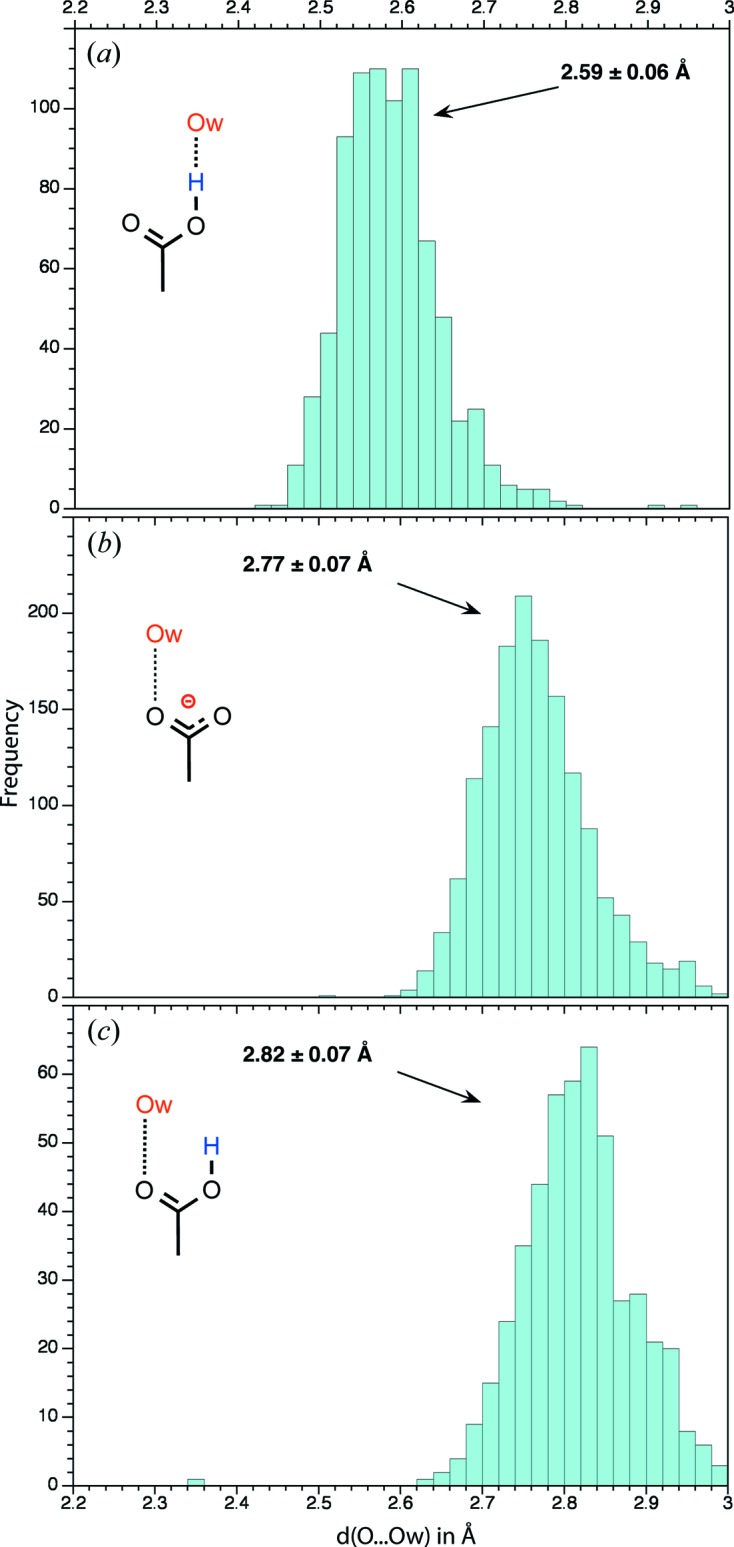
Histograms showing the distance distribution between the two O atoms directly involved in the carboxyl(ate)–water hydrogen bond. For clarity, only water molecules positioned in a 1 Å slice above and below the plane defined by the three heavy atoms of the carboxyl(ate) groups are considered. A cut-off of 2.2 Å for *d*(C=O⋯H—O*w*) or *d*(C—OH⋯O*w*) was used. (*a*) *d*(C—OH⋯O*w*) histogram involving carboxyl groups. (*b*) *d*(C=O⋯O*w*) histogram involving carboxylate groups. (*c*) *d*(C=O⋯O*w*) histogram involving carbonyl O atoms of the carboxyl group.

The hydrogen-bond length between carboxyl(ate) groups and water molecules is strongly dependent on the acceptor or donor character of the former. When bound to the hydroxyl group, the average *d*(C—O(H)⋯O*w*) distance is 2.59 ± 0.06 Å (Fig. 11 ▶ *a*); when bound to a carboxyl(ate) carbonyl group, the average *d*(C=O⋯O*w*) distance (2.77 ± 0.07 Å) becomes close to water hydrogen-bond distances (Figs. 11 ▶ *b* and *c*). The shortest reported hydrogen-bond lengths are close to 2.4 Å. Such a short length is found in the CACTUW structure (Vishweshwar *et al.*, 2004 ▶), where the (C=O)O—H⋯O*w* distance is close to 2.48 Å and involves an *anti* conformer (Fig. 9 ▶ *a*). Interestingly, only 44 water molecules establish a hydrogen bond with the lone pair of the carboxyl—OH group either in *syn* or *anti* (compared with the ∼ 2800 water molecules found around the other groups), confirming its poor acceptor potential. The associated distances are close to 2.80 Å.

#### 3.2.6. Catemers   

**Figure 12:**
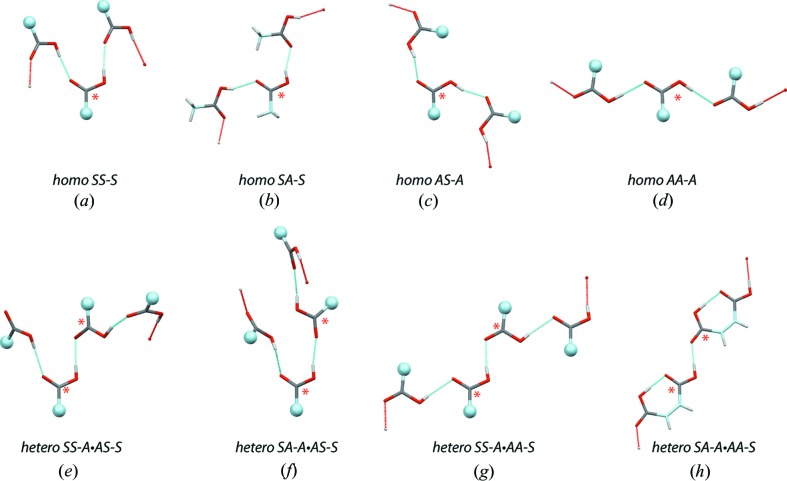
Examples of the eight catemer types identified in the CSD. The C and O atoms not belonging to the interacting carboxyl groups are shown in light blue. The white and red dots mark the position of the connected carboxylic groups in the catemeric chain. The red asterisks mark the carboxyl groups used for naming the catemer. The light blue spheres indicate that the molecule has been truncated for visualization purposes. (*a*) *SS-S* homo-catemer (XONNET); (*b*) *SA-S* homo-catemer (ACETAC07); (*c*) *AS-A* homo-catemer (GIMRAW); (*d*) *AA-A* homo-catemer (DMOXBA01); (*e*) *SS-A·AS-S* hetero-catemer (ROZHEU); (*f*) *SS-A·AA-S* hetero-catemer (WOKPOC); (*g*) *SA-A·AS-S* hetero-catemer (MEKLOE). (*h*) *SA-A·AA-S* hetero-catemer (MALIAC12).

*Nomenclature*: The dimer nomenclature can be adapted without major modifications to the catemer motifs for which two classes can be defined: (i) the *homo-catemers* involving the formation of a continuous chain of the same dimer and (ii) the *hetero*-*catemers* involving two alternating dimer types. In the latter case, we impose the convention that the *syn* conformer precedes the *anti* conformer. Thus, the *SS-A·AS-S* code should be used instead of the *AS-S·SS-A* code. In the current CSD release, four *homo-* and four *hetero-catemer* types were identified (Table 4 ▶ and Fig. 12 ▶).

*Catemer formation rule*: The *SS-S* and *SA-S homo-catemers* are the most represented followed by the *SS-A·AA-S hetero-catemers*. Three other catemers are poorly represented but still present in the CSD. These catemers involve the eight ‘*carbonyl dimers*’ shown Fig. 4 ▶.

After closer examination of the catemer nomenclature (Table 4 ▶), a simple rule emerged. If the dimer starts with a *syn* or an *anti* conformer it should end with an identical conformer. Thus, the *SS-S*, *SA-S*, *AS-A* and *AA-A* dimers form *homo-catemers* since the first and the last conformers are identical, while the *SS-A*, *SA-A*, *AS-S* and *AA-S* dimers need to associate with a complementary motif and can only form *hetero-catemers*. According to this rule, all eight possible *homo-* and *hetero-catemer* combinations were identified in the CSD, although the *SS-A·AS-S* (ROZHEU; Dawid *et al.*, 2009 ▶) and *SA-A·AS-S* catemers (MEKLOE; Das & Desiraju, 2006 ▶) were identified in only one instance. Table S1 of the supporting information provides a list of all characterized catemers, which were manually checked to confirm that they are not part of large rings.

## 4. Discussion   

### 4.1. A systematic classification of carboxyl–carboxyl(ate) dimers…   

By using simple stereochemical considerations, we have demonstrated that the apparently overwhelming diversity of carboxyl–carboxyl(ate) dimers (Rodríguez-Cuamatzi *et al.*, 2007 ▶) can be reduced to 17 supramolecular motifs when considering free rotation around the interlinking hydrogen bond. A hierarchy of motifs emerged that distinguishes first the cyclic dimer (1929 fragment occurrences), followed by the *SS* (947 occurrences), *SA* (357 occurrences) and *SA-S* dimers (234 occurrences) (Table 3 ▶). The other dimers are less represented and some are rare, especially those in the ‘*hydroxyl dimer*’ class where the *as-a* and the *aa-s* types are absent from the current CSD release (Fig. 4 ▶). This latter observation is in agreement with the fact that strong donor groups such as carboxyl —OH functions are also poor acceptors, as reported in small molecules and biomolecular systems (Ramanadham *et al.*, 1993 ▶; Steiner, 2002 ▶).

The reasons as to why in certain circumstances, carboxyl groups prefer to form single hydrogen-bonded dimers extending sometimes into polymeric-like catemeric chains rather than cyclic dimers remains a subject of astonishment, although much has been written on this topic including considerations related to the preferential involvement of *syn* and *anti* lone pairs and conformers (Glusker, 1998 ▶; Sato & Hirata, 1999 ▶; Nagy, 2013 ▶).

In order to appreciate better these conformational preferences, statistical models predicting the number of hydrogen bonds that might form between any donor/acceptor pair in a crystal structure have been derived using CSD data (Allen *et al.*, 1999 ▶; Galek *et al.*, 2014 ▶) along with computational models providing estimates of their intrinsic stability (Dunitz & Gavezzotti, 2012 ▶). These studies confirmed the pre-eminence of the cyclic dimer over other motifs. Although such approaches appear promising, they suffer from: (i) drawbacks related to the still noticeable lack of a sufficient number of crystal structures; (ii) the difficulty to take into account environmental effects; (iii) important approximations in the calculation of the interatomic forces at play in such complex systems. In this respect, non-additive contributions are especially difficult to estimate and quantum mechanical calculations confirmed that the energy gap between different motifs is small and lies within the precision limits of the methods (Meot-Ner *et al.*, 1999 ▶; Meot-Ner, 2012 ▶).

The most important factor to take into account is related to the strong competition of alternate binding motifs. Indeed, in CSD crystal structures, it was established that the probability of formation of dimers was around 30%, the remainder forming hydrogen bonds with a great variety of other acceptors (Steiner, 2001 ▶, 2002 ▶). Interestingly, unforeseen motifs are still brought to light. To cite only a few of them, new crystal forms of aspirin were recently published (Hursthouse *et al.*, 2011 ▶) and a crystallization study of a family of mono-substituted salicylic acid compounds reported an unexpectedly large diversity of motifs (Montis & Hursthouse, 2012 ▶). To understand the association rules of these supramolecular synthons and to be able to be truly predictive, we probably still have to expand current databases by orders of magnitude.

### 4.2. … and associated catemers   

For catemers, we designed a simple rule derived from the carboxyl–carboxyl(ate) dimer nomenclature that postulates that only eight catemer motifs can be formed (Fig. 12 ▶). As for dimers, a catemer hierarchy exists, with the *SA-S* catemer being the most represented (Table 4 ▶). The possible origin of the less frequent formation of catemer motifs over the common cyclic dimer has been addressed by several authors and is of special interest in crystal engineering (Beyer & Price, 2000 ▶; Das & Desiraju, 2006 ▶; Sanphui *et al.*, 2013 ▶). Basically, the same factors involved in the preferential formation of one or the other dimer play a role here, namely steric factors, supporting C—H⋯O interactions and hydrogen-bond competition with various types of chemical groups in addition to specific stereoelectronic effects. These observations stress that intrinsic or local energetic considerations are not sufficient to describe the formation rules of these motifs (Leiserowitz, 1976 ▶; Berkovitch-Yellin & Leiserowitz, 1982 ▶; Kuduva *et al.*, 1999 ▶; Das & Desiraju, 2006 ▶; Hursthouse *et al.*, 2011 ▶).

As for dimers, new catemer patterns are still uncovered such as in the 1,2-phenylenedipropynoic acid where two carboxylic groups from the same molecule are involved in the formation of a *SA-A·AS-S* catemeric chain (unfortunately the structure was not deposited in the CSD; Saravanakumar *et al.*, 2009 ▶). Furthermore, recent examples of carboxylic acid catemer and dimer synthon polymorphs were reported (Gajda *et al.*, 2009 ▶; Sanphui *et al.*, 2013 ▶). Overall, we characterized 122 catemers that can be compared with the 73 catemers characterized from a survey of the April 1998 CSD (Kuduva *et al.*, 1999 ▶). Note that in this present study, we were able to categorize two particularly rare catemers observed in only one instance each (Table 4 ▶). This is fortunate since we believe to have now a complete structural sample of each of the eight possible homo- and hetero-catemer structures.

### 4.3. Short hydrogen bonds   

Besides these classification attempts, this study supports findings established in earlier surveys on smaller structural samples that hydrogen bonds involving carboxyl–carboxylate dimers are on the shorter and consequently stronger side of hydrogen bonds (Jeffrey & Saenger, 1991 ▶; Jeffrey, 1997 ▶; Steiner, 2001 ▶, 2002 ▶; Vishweshwar *et al.*, 2004 ▶; Langkilde *et al.*, 2008 ▶). It is beyond the scope of this paper to analyse the reasons as to why such short hydrogen bonds are formed. However, the topic of short or ‘strong’ hydrogen bonds involving amongst others the carboxyl(ate) groups found in proteins has received great attention especially since they were associated with enzymatic catalytic mechanisms (Perrin & Nielson, 1997 ▶; Katz *et al.*, 2002 ▶; Gilli & Gilli, 2009 ▶; Perrin, 2010 ▶; Hosur *et al.*, 2013 ▶) involving either the *syn* or *anti* lone pairs (Zimmerman *et al.*, 1991 ▶).

The carboxyl–carboxyl hydrogen bonds are generally considered as π-cooperative bonds or bonds belonging to the class of ‘resonance-assisted hydrogen bonds’ (RAHB; Vishweshwar *et al.*, 2004 ▶; Bertolasi *et al.*, 2006 ▶; Gilli & Gilli, 2009 ▶). In these motifs, the COOH donor is activated by π-cooperative hydrogen bonding (O—H⋯O=C). The carboxyl–carboxylate hydrogen bonds that involve a bond between an acid and its conjugate base fall clearly in a different pool where the stabilizing effect is induced by the presence of the negative charge. These bonds are also called ionic hydrogen bonds (Steiner, 1999 ▶; Meot-Ner, 2012 ▶) or negatively ‘charge-assisted hydrogen bonds’ (CAHB; Vishweshwar *et al.*, 2004 ▶; Gilli & Gilli, 2009 ▶). They are on average ∼ 0.1 Å shorter than the RAHB hydrogen bonds (Fig. 6 ▶). This is particularly obvious when both groups have similar p*K*_a_ values as in protein structures where they play important structural and sometimes catalytic functions (Cleland & Kreevoy, 1994 ▶; Hosur *et al.*, 2013 ▶).

A third category of hydrogen bonds is found in mono-anion dicarboxylic compounds (Fig. 3 ▶). These intramolecular hydrogen bonds can be regarded as very short CAHBs given their average 2.43 Å distance (Fig. 6 ▶ *d*). Consequently, they also belong to the strongest class of hydrogen bonds among those involving carboxyl(ate) groups. The shortening of the hydrogen bond is attributed to the presence of the electronegative O acceptor atom. They are probably further stabilized by some synergism due to increased π-delocalization facilitated by their intramolecular character (Perrin & Nielson, 1997 ▶). These dimers involve both the *anti* conformer and a carbonyl lone pair, supporting the view that the lone pair basicity scale might be essentially contextual. Further, these mono-anion dicarboxylic compounds are involved in the formation of at least two types of hetero-catemeric chains: (i) the *SA-A·AS-S* (Fig. 7 ▶ *d*) and (ii) *SA-A·AA-S* types (Fig. 13*d*).

Rather counterintuitively, the shortest carboxyl(ate)–water hydrogen bonds involve the neutral carboxyl and not the charged carboxylate group (Fig. 11 ▶). Such short hydrogen bonds were analysed by density functional theory (Śmiechowski *et al.*, 2011 ▶; Brown *et al.*, 2012 ▶) and extensively discussed in a small-molecule neutron diffraction study where the authors were able to demonstrate the associated chain of polarization events (Vishweshwar *et al.*, 2004 ▶). The latter group observed that not only charge and resonance assistance can lead to very short intermolecular hydrogen bonds [*d*(O⋯O) ≃ 2.4–2.5 Å], but polarization assistance must also be considered in terms of σ-cooperative stabilization (see Fig. 9 ▶ *a*). These synergistic effects were named ‘synthon-assisted hydrogen bonds’ or SAHB (Brown *et al.*, 2012 ▶). Examples of such multi-centred short hydrogen bonds can also be found in biomolecular systems and might play a significant role at catalytic sites (Cleland & Kreevoy, 1994 ▶; Katz *et al.*, 2002 ▶).

### 4.4. Implications for biomolecular systems   

Carboxyl dimers that involve simultaneous protonation of two Asp/Glu amino acids have not been reported in biomolecular systems, although carboxyl–carboxylate dimers appear to be relatively frequent in a wide pH range that can extend to 8.0 (Sawyer & James, 1982 ▶; Flocco & Mowbray, 1995 ▶; Torshin *et al.*, 2003 ▶; Wohlfahrt, 2005 ▶; Langkilde *et al.*, 2008 ▶). The formation of such interactions is surprising since it is generally assumed that given the p*K*_a_ of the Asp (∼ 3.9) and Glu (∼ 4.3) residues (Pace *et al.*, 2009 ▶), they would be deprotonated at physiological pH. As an outcome, carboxyl(ate) groups can form four different dimer types that extend to 16 when the two Asp/Glu amino-acid types are considered. However, since H-atom positions can rarely be observed in macromolecular systems, *SA* and *AS* dimers cannot be differentiated and this number reduces to nine due to degeneracy.

It was reported that the *SA*/*AS* arrangement is the most common in proteins (62%) followed by *SS* (24%) and *AA* (14%; Wohlfahrt, 2005 ▶), in contrast to the present study where the *SS* dimer dominates (Table 3 ▶). This originates probably from the better accessibility of the *anti* lone pairs of the Asp/Glu residues that are not shielded by large chemical groups, as is observed in a majority of CSD structures. However, it remains to be determined whether the *SA* or *AS* arrangements is favoured or if they are energetically not differentiable. In other words, if the *anti* conformer is preferred or not over the *syn* conformer or if these preferences are contextual as so often witnessed in all types of chemical systems. Theoretical calculations on model systems favour the *AS* arrangement (Wohlfahrt, 2005 ▶), while the present study identifies the *SA* arrangement as being the most frequent (Table 3 ▶).

To identify the protonated state of Asp/Glu residues in X-ray structures, efforts based on stereochemical factors have been made. The most obvious consideration relates to the hydrogen-bond proximity of two carboxyl(ate) O atoms, the associated distance being generally well below 2.7 Å (Sawyer & James, 1982 ▶; Ramanadham *et al.*, 1993 ▶; Flocco & Mowbray, 1995 ▶; Torshin *et al.*, 2003 ▶; Wohlfahrt, 2005 ▶; Langkilde *et al.*, 2008 ▶). The carboxyl C—O(H) and C=O bond lengths differ by ∼ 0.1 Å (Table 2 ▶) and the bond electron densities have also been exploited in the analysis of high-resolution protein structures (≤ 1.3 Å), leading to clear identification of protonated Asp/Glu residues (Ahmed *et al.*, 2007 ▶; Fisher *et al.*, 2012 ▶). In the absence of good neutron diffraction structures (Ahmed *et al.*, 2007 ▶; Hosur *et al.*, 2013 ▶), such techniques could help to unscramble the degeneracy issue mentioned above. On a similar line of thought, short side-chain Asp/Glu carboxyl(ate) to O*w* distances could be used to infer protonation states of the residues (Ramanadham *et al.*, 1993 ▶).

## 5. Summary and perspectives   

This work illustrates the diversity of supramolecular motifs generated by a single chemical group and offers a comprehensive carboxyl–carboxyl(ate) dimer and catemer nomenclature. As noted above:

(i) 17 possible carboxyl–carboxyl(ate) interaction modes including *syn* and *anti* conformers as well as carbonyl lone pairs were identified;
(ii) among them, the cyclic dimer is the most represented;
(iii) instances of all other possible interaction modes were found in the CSD, except the two *as-a* and *aa-s* ‘*hydroxyl dimers*’;
(iv) based on this classification, eight catemeric types could be uniquely identified;
(v) the *anti* conformers are well represented and form distinguishable supramolecular motifs implying no significant basicity difference between the *syn* and *anti* lone pairs;
(vi) the strongest (intramolecular) hydrogen bonds are observed in mono-anion dicarboxylic compounds and involve simultaneously an *anti* conformer and an *anti* lone pair, supporting the fact that *anti* interactions are by no means weaker than *syn* interactions;
(vii) the shortest hydrogen-bond lengths found in this survey, including those formed with water molecules, are close to 2.36 Å (Fig. 6 ▶ *d*);
(viii) cooperative effects appear to be important in probably all systems involving carboxyl(ate) groups and should always be considered.

Although significant progress has been achieved in crystal engineering, it seems appropriate to recall a sobering thought by Steiner, who wrote in a paper on hydrogen-bond competition: ‘*Even though it is true that strong hydrogen-bond donors tend to interact with strong acceptors, this is valid only as a tendency. Weak acceptors also have a certain chance of attracting the strong donor. This weakens the general applicability of rules for predicting hydrogen-bond modes from hierarchies of donor and acceptor strengths and indeed all such rules published are very unreliable in practice*’ (Steiner, 2001 ▶). Further, Desiraju, witnessing the constant discovery of unforeseen structures, noted that after all: ‘*it would seem that brute-force method will eventually win*’ (Desiraju, 2007 ▶), suggesting that many more interaction rules of increasing complexity will be brought to light and that concerted but also serendipitous crystallization experiments are still very much needed to make progress in the field. These considerations on small supramolecular synthons apply fully to biomolecular systems where carboxyl(ate) groups are found to adapt in surprising and still insufficiently documented ways to their local environment.

## Supplementary Material

Complete list of CSD codes relating to catemer structures. DOI: 10.1107/S205252061500270X/bi5041sup1.pdf

## Figures and Tables

**Table 1:** Number of structures in the CSD (Version 5.35, November 2013) containing at least one carboxyl, carboxylate or metal-bound carboxyl(ate) group and number of structures with low *R*-factor values (*R* 0.05). Disordered, error-containing, polymeric and powder structures were excluded from the search

	All	*R* 0.05
Carboxyl	14452	8254
Carboxylate	9283	5446
Metal-bound carboxyl	492	305
Metal-bound carboxylate	13438	9082
Total	37665	23087

**Table 2:** Number of structures and fragments containing carboxyl(ate) groups in the CSD Only low *R*-factor structures (*R* 0.05) are considered. Statistics were also collected for the *anti* conformer subgroups that take into account the carboxyl groups that are involved in intra- and intermolecular hydrogen bonds, respectively. Distances are in , angles in .

	No. of structures	No. of fragments	*d*(CO)	*d*(CO)	(OCO)	(CCO)	(CCO)	(CCOH)
Carboxyl-*syn*	6852	9295	1.22 0.02	1.31 0.02	124 1	123 2	113 2	111 3
Carboxyl-*anti* (intermolecular)	209	223	1.21 0.01	1.31 0.02	121 2	122 2	117 2	112 4
Carboxyl-*anti* (intramolecular)	760	945	1.22 0.01	1.30 0.02	121 1	120 2	118 2	110 4
Carboxylate	5353	6760	1.25 0.02		125 2	117 2		

**Table 3:** Number of structures and fragments containing carboxylcarboxyl(ate) dimers in the CSD

	No. of structures†	No. of fragments†	*d*(OO)‡
Carboxylcarboxyl			
*Cyclic dimer*	1741 (2984)	1929 (3385)	2.65 0.03
			
Carbonyl dimer			
*SS-S*	57 (91)	64 (98)	2.68 0.04
*SS-A*	57 (80)	62 (88)	2.64 0.05
*SA-S*	204 (333)	234 (378)	2.67 0.05
*SA-A*	18 (25)	19 (26)	2.65 0.06
*AS-S*	4 (6)	4 (6)	2.68 0.05
*AS-A*	6 (7)	6 (7)	2.64 0.02
*AA-S*	11 (15)	11 (16)	2.64 0.04
*AA-A*	3 (3)	3 (3)	2.70 0.04
			
Hydroxyl dimer			
*ss-a*	2 (7)	2 (7)	2.71
*sa-s*	6 (8)	6 (8)	2.76 0.12
*as-a*	()	()	
*aa-s*	()	()	
			
Carboxylcarboxylate			
*SS*	801 (1199)	947 (1429)	2.53 0.05
*SA*	319 (492)	357 (554)	2.58 0.05
*AS*	27 (48)	29 (52)	2.52 0.06
*AA*	61 (102)	68 (117)	2.54 0.06

† The number of structures and fragments are given for structures with low *R*-factors (*R* 0.05). The number of structures and fragments derived from the entire CSD (no *R*-factor restrictions) are given in parentheses.

‡ Average distances () calculated for the *R* 0.05subset.

**Table 4:** Number of catemer-containing structures in the CSD Only low *R*-factor structures (*R* 0.05) are taken into account (see complete list in Table S1). Disordered, error-containing, polymeric and powder structures were excluded from the search.

	No. of structures
Homo-catemer	
*SS-S*	23
*SA-S*	67
*AS-A*	3
*AA-A*	3
	
Hetero-catemer	
*SS-AAS-S*	1
*SS-AAA-S*	17
*SA-AAS-S*	1
*SA-AAA-S*	7
