# Supplementary material for: A comprehensive classification and nomenclature of carboxyl–carboxyl(ate) supramolecular motifs and related catemers: implications for biomolecular systems
Source: Acta Crystallogr B Struct Sci Cryst Eng Mater. 2015 Mar 24;71(Pt 2):164–75. doi: 10.1107/S205252061500270X (PMC4383392; doi:10.1107/S205252061500270X)
Supplement: Supplementary file 1 [file b-71-00164-sup1.pdf]

STRUCTURAL SCIENCE
CRYSTAL ENGINEERING
MATERIALS

Volume 71 (2015)

Supporting information for article:

A comprehensive classification and nomenclature of carboxyl–carboxyl(ate) supramolecular motifs and related catemers: implications for biomolecular systems

Luigi D'Ascenzo and Pascal Auffinger

Table S1 List of all catemer-containing structures identified in the CSD with $R1 \leq 0.05$.

Homo-catemer	
SS-S	DAYNUN; ENIFAJ; FIKJEO; GUWCOQ; HUKJUT; HUSXAU; IBUHES; IBUHOC; MUCQAD; NAGVUM; SUVYEN; TARTAC; TARTAL; TARTAL01; TARTAL02; TARTAL03; TARTAL04; TORTEA; VAFCUB; WOCHIF; XEDZUC; XONNET; ZOGTUK
SA-S	ACETAC07; ACETAC09; ARUVAK; BELQOZ; BIPCIQ10; BUJYEL; CANSAL; CIJLOW; CIPZIL; CITRAC10; CITRAC11; CLACET01; DIYDIY; DMCPCX; DOTWOY; EVORIQ; EYONAI; FIWQEI; FOHREI; FORMAC01; GELZIG; GOGPEY; GOGPIC; HEKWOJ; HIFWOJ; HUGSEH; HUWLIV; IMEHIS; IROQOV; ISORUD; ISOSAK; ISOSAL; IVEBIV; JUMVIW; KABGOL; KIKLIZ; KOJZEO; KUTMIW; MEKMOF; MIYCON; MEDNOZ01; NUFJUU; OWUSEF; OXALAC05; OXALAC07; PEPPAD; PEZWAS; QAGMOG; QUQHAL; QURQID; RACCEE; SAWBUN; SDPPCX; TEHMAU; TETROL01; TUSPOM; UCAYUU; URUPUT; VEVSIZ; VOHNUC; WANROU; WASJAD01; WINVAR; WOCTUD; WUXHUS; ZAVTOF; ZILBOL
AS-A	GIMRAW; NEWXAO; ROGHOL
AA-A	DMOXDA01; DMOXDA02; HUMGOL
Hetero-catemer	
SS-A•AS-S	ROZHEU
SS-A•AA-S	BCOCDC; CBUDCX; CBUDCX01; CBUDCX02; CBUDCX03; CBUDCX04; COMHFN; COMHFN01; CPRDCA; FIGMAJ; FURDCB01; IDAKAB; JUNCUQ; JUNMAG; MEKLOE; MIGPEX; RAJJUH
SA-A•AS-S	WOKPOC
SA-A•AA-S	CPIBFC; CUKGIZ; KAMKAK; KAKTOS; MALIAC11; MALIAC12; MALIAC13
